# Supplementary material for: Interplay of water and a supramolecular capsule for catalysis of reductive elimination reaction from gold
Source: Nat Commun. 2020 Jan 21;11:415. doi: 10.1038/s41467-019-14251-6 (PMC6972886; doi:10.1038/s41467-019-14251-6)
Supplement: Supplementary file 1 — Supplementary Information [file 41467_2019_14251_MOESM1_ESM.docx]

**An Encapsulated Water Governs Catalysis in a Supramolecular System for Reductive Elimination Reaction from Gold**

Vaissier Welborn et al.

**SUPPORTING INFORMATION**

**An Encapsulated Water Governs Catalysis in a Supramolecular System for Reductive Elimination Reaction from Gold**

Valerie Vaissier Welborn, Wan-Lu Li, and Teresa Head-Gordon

**Reactant State Transition State**


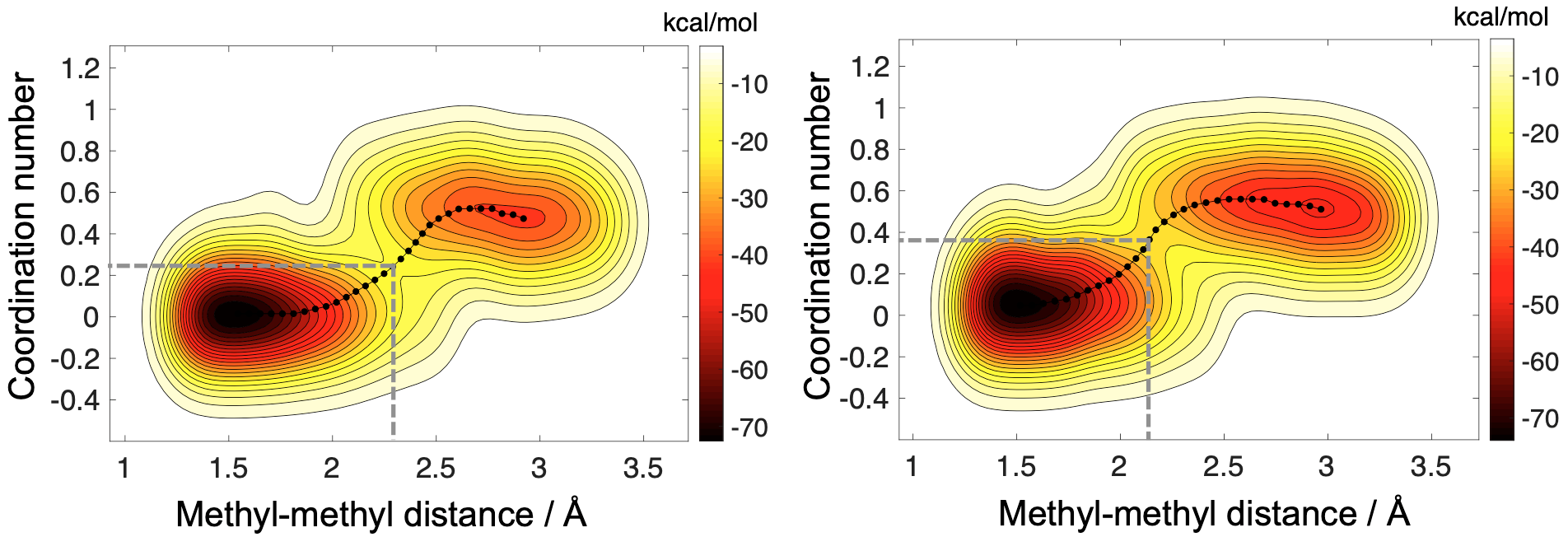


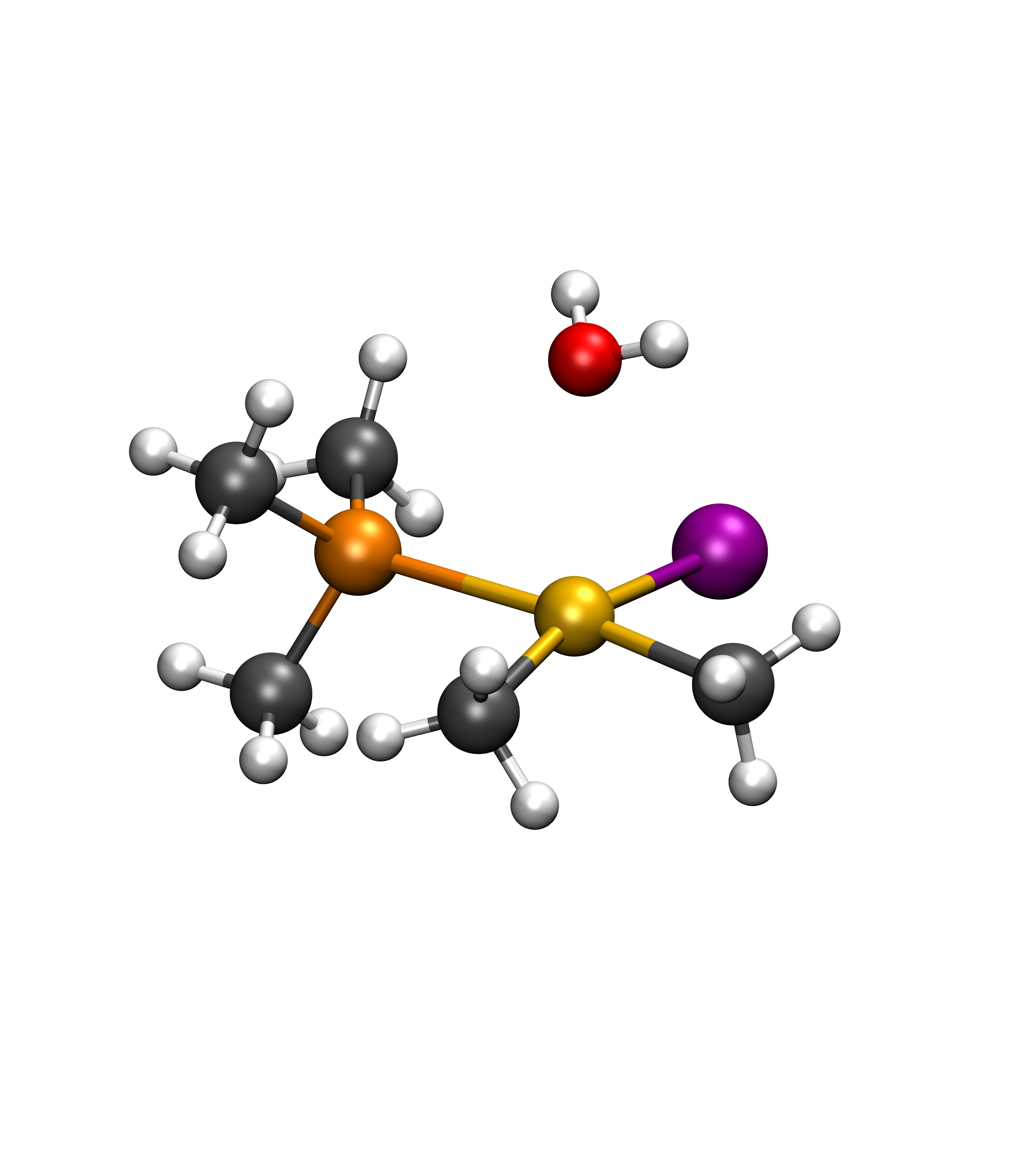


3.7 Å


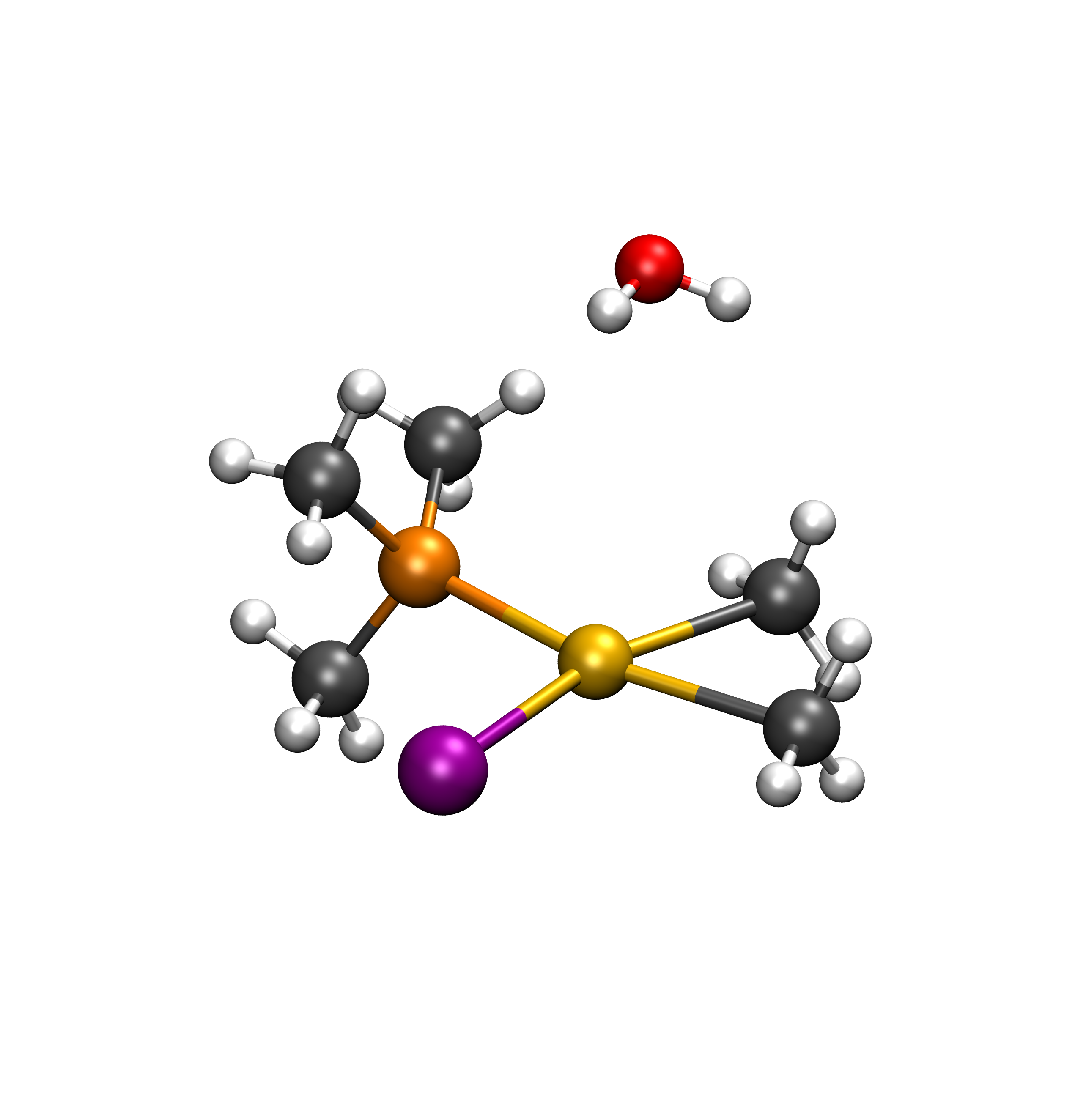


4.3 Å

**Supplementary Figure 1.** *Free energy landscape obtained from ab initio metadynamics for the halogenated P(CH_3_)_3_(CH_3_)_2_AuI in water in the absence of the cage.* The transition state was located with the string method to be at (2.3 Å, 0.24).

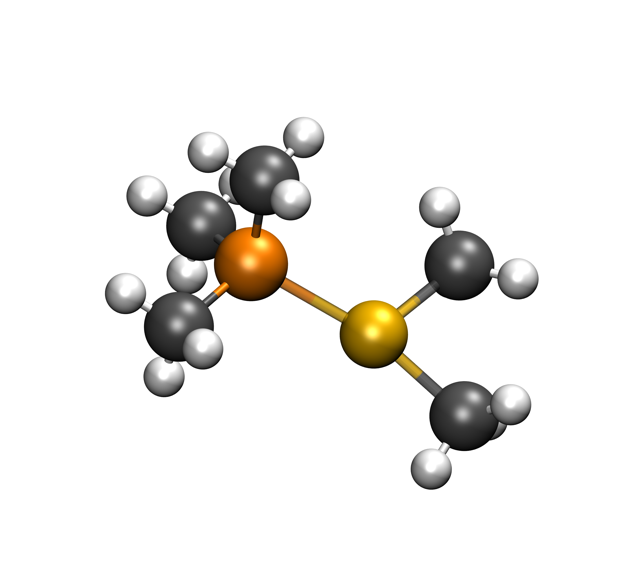


**Supplementary Figure 2.** *Bond dipoles considered in this work.*

The dipoles were computed from the partial charges on the gold and carbon atoms (c_Au_ and c_Ci_ in the formula above), the bond length d_Au-Ci_ and conversion factor (CF=1/0.208194) to get the dipole in Debye.

**Supplementary Table 1.** *Density derived atomic charges and bond dipoles for the uncatalyzed and catalyzed reactions.* Charges for the gold and carbon (of the methyl groups) atoms and given the bond lengths the corresponding bond dipoles in reactant and transition state. The dipoles are given in Debye with the corresponding bond length in Å.

| Reaction type | Atoms | RS Charges | TS Charges | Bond Type | RS Bond Dipole (bond length) | TS Bond Dipole (bond length) |
| --- | --- | --- | --- | --- | --- | --- |
| Uncatalyzed reaction with iodide | Au | 0.18895 | 0.102974 | Au-C1  Au-C2 | -3.96 D (2.09 Å)  1.65 D (2.09 Å) | -3.37 D (2.54 Å)  1.24 D (2.52 Å) |
|  | C1 | -0.205939 | -0.173502 |  |  |  |
|  | C2 | 0.353906 | 0.205655 |  |  |  |
| Uncatalyzed reaction without iodide | Au | 0.352889 | 0.136622 | Au-C1  Au-C2 | -6.15 D (2.07 Å)  -2.83 D (2.07 Å) | -1.26 D (2.4 Å)  3.67 D (2.3 Å) |
|  | C1 | -0.266251 | 0.026892 |  |  |  |
|  | C2 | 0.068112 | 0.47181 |  |  |  |
| Catalyzed reaction | Au | 0.352889 | 0.121254 | Au-C1  Au-C2 | -6.15 D (2.07 Å)  -2.83 D (2.07 Å) | 2.05 D (2.52 Å)  0.71 D (2.58 Å) |
|  | C1 | -0.266251 | 0.290602 |  |  |  |
|  | C2 | 0.068112 | 0.178907 |  |  |  |
